# A quasi-randomised controlled trial of online distribution of home-based hepatitis C self-testing for key populations in Malaysia: a study protocol

**DOI:** 10.1186/s13063-022-06230-y

**Published:** 2022-04-12

**Authors:** Sonjelle Shilton, Xiaohui Sem, Huan-Keat Chan, Han Yang Chung, Anu Karunanithy, Jessica Markby, Po-Lin Chan, Niklas Luhmann, Cheryl Johnson, Pamela Nabeta, Nazrila Hairizan Bt Nasir, Stefano Ongarello, Elena Ivanova Reipold, Muhammad Radzi Abu Hassan

**Affiliations:** 1 FIND, Geneva, Switzerland; 2 Clinical Research Centre, Hospital Sultanah Bahiyah, Alor Setar, Malaysia; 3 Drugs for Neglected Diseases initiative South-East Asia Regional Office, Kuala Lumpur, Malaysia; 4 Malaysian AIDS Council, Kuala Lumpur, Malaysia; 5 World Health Organization Regional Office for Western Pacific, Manila, Philippines; 6 Global HIV, Hepatitis & STI Programmes, World Health Organization, Geneva, Switzerland; 7 Family Health Development Division, Ministry of Health, Putrajaya, Malaysia

**Keywords:** Hepatitis C, Self-testing, Key populations, Malaysia, Testing uptake, Linkage to care

## Abstract

**Background:**

Malaysia has an estimated hepatitis C virus (HCV) prevalence of 1.9% among its adult population and a history of providing HCV treatment in the public sector. In 2019, Malaysia launched a 5-year national strategic plan for viral hepatitis control and has been expanding HCV testing and treatment to the primary care and community levels, while actively engaging key populations in services for hepatitis care. The Ministry of Health (MoH) is seeking to specifically understand how to better target HCV services at men who have sex with men (MSM); HCV self-testing could increase the uptake of HCV testing among this group.

**Methods:**

We aim to integrate HCV antibody self-testing into an existing online platform used for HIV self-testing, to evaluate the acceptability and impact of an online HCV self-testing programme in Malaysia. This is a non-blinded parallel group quasi-randomised superiority study comparing HCV self-testing via an online distribution model with the standard care, which involves attending a clinic for facility-based HCV antibody testing (control, 2:1). Participants will be randomised to either the HCV self-testing via online distribution arm, in which either an oral fluid- or blood-based HCV self-test kit will be mailed to them, or the control arm, where they will be provided with information about the nearest centre with HCV testing. The primary outcome is the number and proportion of participants who report completion of testing. Secondary outcomes include the number and proportion of participants who (a) receive a positive result and are made aware of their status, (b) are referred to and complete HCV RNA confirmatory testing, and (c) start treatment. Acceptability, feasibility, attitudes around HCV testing, and cost will also be evaluated. The target sample size is 750 participants.

**Discussion:**

This study is one of the first in the world to explore the real-world impact of HCV self-testing on key populations using online platforms and compare this with standard HCV testing services. The outcomes of this study will provide critical evidence about testing uptake, linkage to care, acceptability, and any social harms that may emerge due to HCV self-testing.

**Trial registration:**

ClinicalTrials.gov NCT04982718

**Supplementary Information:**

The online version contains supplementary material available at 10.1186/s13063-022-06230-y.

## Administrative information

Note: The numbers in curly brackets in this protocol refer to SPIRIT checklist item numbers. The order of the items has been modified to group similar items (see http://www.equator-network.org/reporting-guidelines/spirit-2013-statement-defining-standard-protocol-items-for-clinical-trials/).

**Table:** 

Title {1}	Randomised controlled trial of online distribution of home-based hepatitis C self-testing for key populations in Malaysia
Trial registration {2a and 2b}	ClinicalTrials.gov ID NCT04982718
Protocol version {3}	V 3.0 15 December 2021
Funding {4}	The Government of the Netherlands
Author details {5a}	1. FIND, Geneva, Switzerland 2. Clinical Research Centre, Hospital Sultanah Bahiyah, Alor Setar, Malaysia 3. Drugs for Neglected Diseases *initiative* South-East Asia Regional Office, Kuala Lumpur, Malaysia 4. Malaysian AIDS Council, Kuala Lumpur, Malaysia 5. World Health Organization Regional Office for Western Pacific, Manila, Philippines 6. World Health Organization, Global HIV, Hepatitis & STI Programmes, Geneva, Switzerland 7. Family Health Development Division, Ministry of Health, Putrajaya, Malaysia
Name and contact information for the trial sponsor {5b}	FIND, Sonjelle Shilton, Sonjelle.shilton@finddx.org
Role of sponsor {5c}	The funders have played no role in the study design and will play no role in the collection, management, analysis or interpretation of the data; writing of the report; or decision to submit the report for publication. The sponsor, FIND, in collaboration with implementing partners, wrote the protocol and will play a role in the collection, management, analysis and interpretation of the data; writing of the report; and decision to submit the report for publication. The findings and conclusions in this report are those of the authors and do not necessarily represent the official position of the WHO.

## Introduction

### Background and rationale {6a}

Globally, only 21% of the estimated 58 million people living with hepatitis C virus (HCV) know their HCV status [1]. To achieve the 2030 World Health Organization (WHO) global goals for hepatitis elimination, a significant scale-up of testing for hepatitis is needed [2]. HCV self-testing (referring to serological testing for HCV antibodies) could be a valuable tool that contributes to this scale-up of testing. WHO recently produced the first-ever recommendations on HCV self-testing [3]. These recommendations leveraged the large evidence base that has accumulated on the value of HIV self-testing as well as data from various studies on the feasibility, acceptability and values and preferences around HCV self-testing [4–8]. This evidence base demonstrates that people are able to correctly perform and interpret the results of an HCV self-test and that HCV self-testing is feasible and acceptable to end-users. The new WHO recommendations do note, however, that information is needed about how HCV self-testing impacts the uptake of HCV testing and linkage to care and how it is best delivered.

Self-testing for HIV has been shown to increase testing rates and acceptability in diverse populations around the world, in large part due to its convenience and privacy advantages [9]. However, the health systems in place for HIV are much more extensive than those which currently exist for HCV. Whereas testing and treatment for HIV are widely available through the public sector globally, the number of countries that currently provide HCV testing and treatment remains relatively limited. There is therefore a need to build an evidence base for the impact of HCV self-testing that includes data relating to uptake, linkage to care, and acceptability, as well as to capture information about any social harms that may emerge due to the use of HCV self-testing.

Malaysia has an estimated HCV prevalence of 1.9% among its adult population according to an opt-in screening campaign conducted in 2019 [10] and has a history of providing hepatitis C treatment in the public sector [11]. In 2019, Malaysia launched a 5-year national strategic plan for viral hepatitis control [12], and the country has been expanding HCV testing and treatment access to the primary care and community levels while actively engaging key populations in services for hepatitis care [11]. One group that the Malaysian Ministry of Health (MoH) would like to understand how to reach more effectively is men who have sex with men (MSM). Malaysia has a strong network of primary health clinics, which serve a large percentage of the population. However, due to considerable socioreligious stigma in Malaysia, MSM may be less likely than other population groups to regularly attend primary health services and therefore represent good candidates for HCV self-testing [13]. To explore the usefulness of HIV self-testing in Malaysia to reach key populations such as MSM, a pilot study offering HIV self-testing via an online platform (JomTest) was launched in November 2020, in partnership with the Malaysian AIDS Council (MAC) and the University of Malaya [14]. JomTest caters to people who identify as MSM, people who inject/use drugs (PWI/UD), transgender women, sex workers and persons engaging sex workers. These same groups may also be at high risk of contracting HCV; however, estimates for HCV prevalence among these groups in Malaysia are currently limited.

In the present study, we will integrate HCV self-testing with the existing online HIV self-testing platform, JomTest. We aim to evaluate the impact, acceptability, feasibility and cost of home-based HCV self-testing made accessible via an online platform in Malaysia.

### Objectives {7}

The primary objective of this study is to assess the impact of home-based HCV self-testing offered via an online platform on the uptake of HCV antibody testing among key populations. The secondary objectives of the study are as follows:

1. To assess the impact of HCV self-testing on the number of HCV antibody-positive individuals who are aware of their HCV status
2. To assess the impact of HCV self-testing on linkage to care and completion of HCV RNA confirmatory testing among HCV antibody-positive individuals
3. To assess the impact of HCV self-testing on treatment initiation in HCV RNA-positive individuals eligible to begin treatment
4. To assess the acceptability and feasibility of HCV self-testing at baseline and after study participation
5. To compare the cost of HCV self-testing with the cost of standard of care

### Trial design {8}

This is a parallel-group non-blinded superiority study comparing HCV self-testing via an online distribution model on the JomTest platform with the standard of care (control). The participant allocation ratio will be 2:1. Participants in the control group will be offered an HCV self-test if they do not go to a primary healthcare clinic for facility-based testing after the completion of two follow-up surveys.

## Methods: participants, interventions and outcomes

### Study setting {9}

This study will be performed nationwide across Malaysia. Due to the home-based nature of self-testing, there are no intervention sites. Participants in the control group will be directed to their nearest clinic that offers facility-based HCV testing.

### Eligibility criteria {10}

Participants are only eligible to be included in the study if all of the following inclusion criteria are met:

- *Age* ≥ 18 years
- *Eligible to access services on the JomTest online platform*
- *Able to read and understand Bahasa Malaysia or English*
- *Able to understand the scope of the study and provide written informed consent*
- Not tested for HCV within the last 6 months

Participants are excluded from the study if they s*elf-report a previously confirmed, positive HCV status (either antibody or RNA test).*

### Who will take informed consent? {26a}

The consenting process will be performed via the online platform. An online form will explain the nature of the study to prospective participants. A hotline will also be available to provide answers to any questions about the study.

Participants will only be able to advance to the next step of the study on the online platform once they have completed the online informed consent form.

### Additional consent provisions for collection and use of participant data and biological specimens {26b}

Not applicable, as this study will not be collecting or using biological specimens.

### Interventions

#### Explanation for the choice of comparators {6b}

As this study is intended to demonstrate the superiority of HCV self-testing compared with the standard of care facility-based testing, this was chosen as the comparator.

#### Intervention description {11a}

Participants will be randomised to the intervention or control groups in a 2:1 ratio. In the intervention group, participants will receive an HCV self-test kit delivered in non-identifiable packaging to their home or a preferred mailing address. The kit will include the test itself, instructions for use, additional supporting materials and information on how to access a live chat facility and a call centre in case they have any questions about the test. To evaluate the two sampling methods for HCV self-testing, the first 250 participants in the intervention group will receive an oral fluid-based HCV self-test, and the next 250 participants will receive a blood-based fingerstick HCV self-test. For the oral fluid-based test, participants must swab their upper and lower gums with the test device, place it in the tube provided and read the results within 20 to 40 min of taking the test. For the blood-based test, participants must collect a drop of blood by pricking a finger with a lancet, transfer the blood onto the test device, add the diluent and read the results after 15 min. Participants will be invited to conduct the HCV self-test and then report back if they completed the test as well as enter the test results via the online platform.

In the control group, participants will receive information about the standard of care HCV antibody testing available at local testing sites in their community and information about additional supporting materials, such as access to the live chat facility and the call centre for any questions about testing. At local testing sites, HCV testing involves obtaining blood via a finger prick or obtaining blood from the arm.

As there is currently no quality-assured HCV self-test available, the professional-use OraQuick® HCV Rapid Antibody Test and First Response® HCV Card Test, which have been adapted by the manufacturers for self-testing by being repackaged and labelled with instructions for use, will be used in this study. The OraQuick® HCV Rapid Antibody Test is prequalified by WHO and CE-marked for professional use (sensitivity 98.1%, specificity 99.6%); the First Response® HCV Card Test is CE-marked and currently under WHO review for professional use (sensitivity 100%, specificity 100%). Neither of these professional-use tests are yet registered with the Medical Device Authority in Malaysia. The OraQuick® HCV Self-Test and First Response® HCV Self-Test kits provided to participants in the intervention group will be labelled for Research Use Only (RUO). For these reasons, HCV self-testing results from this study will not be used for clinical decision making, and all participants who report a positive HCV self-test and who report to a clinic will receive follow-up HCV rapid testing in the clinic.

#### Criteria for discontinuing or modifying allocated interventions {11b}

This is not applicable for this study given its design.

#### Strategies to improve adherence to interventions {11c}

Participants will be provided with several supporting tools to minimise the rate of errors in the self-testing process and any possible confusion in the interpretation of the test results. Printed instructions for use in English and Bahasa Malaysia will be delivered along with the test kit; pictorial guides on how to use the test will also be included. Participants will also be provided with a link to a video guide and have access to a live chat facility and a call centre.

#### Relevant concomitant care permitted or prohibited during the trial {11d}

There is no concomitant care prohibited during the study.

#### Provisions for post-trial care {30}

Participants who are HCV antibody-negative will be encouraged to seek retesting at a later date, as per the recommended testing frequencies for key populations of the Malaysian national HCV algorithm. Any participants found to be HCV antibody-positive through the study will be supported to access HCV management and care services for further HCV testing and HCV treatment, as required by the national HCV programme. Participants will also be provided with additional assistance to facilitate linkage to care through MAC’s existing peer navigation system, as outlined in the organisation’s standard operating procedures.

### Outcomes {12}

The primary outcomes are the number and proportion of participants who report completing HCV antibody testing in the intervention group and to assess whether the proportion of participants who report completing HCV antibody testing in the intervention group is greater than that of the participants in the control group by a margin of 20%.

The secondary outcome measures are as follows: the number and proportion of HCV antibody-positive individuals made aware of their HCV status in the intervention versus the control groups. The number and proportion of HCV antibody-positive individuals who are referred to and complete HCV RNA confirmatory testing in the intervention versus the control groups. The number and proportion of HCV RNA-positive individuals who begin treatment in the intervention versus the control groups. The completion of the Likert scale rankings of ease of use that the participant report regarding the HCV testing process. The cost per test completed and the cost per person diagnosed (serology, RNA) in the intervention versus the control groups.

#### Participant timeline {13}

The participant timeline is presented in Table 1.

**Table 1:** Participant timeline

Procedure	Enrolment	Test results	Follow-up #1	Follow-up #2
	Day 0	Weeks 2–4	Weeks 2–4	Weeks 4–8
Inclusion and exclusion criteria via the online survey	X			
Informed consent via the online form	X			
Demographic information; Knowledge, Attitudes and Practices (KAP) survey; risk behaviour via the online survey	X			
Completion of follow-up survey #1 regarding the perceptions of testing processes, uploading of test results, KAP and further linkage to care (if applicable)		X	X	
Completion of follow-up survey #2 regarding the risk behaviours and further linkage to care (if applicable)				X
Linking of data of RNA test results and treatment initiation (if applicable)			X	X
Adverse events/severe adverse events/social harms review	X	X	X	X
Cost data collection/analysis	X	X	X	X

#### Sample size {14}

The study will enrol a minimum of 750 participants who meet the inclusion criteria. There will be a minimum of 250 participants who receive an oral fluid-based HCV self-test, 250 participants who receive a blood-based HCV self-test and 250 participants in the control group. The study is powered to detect at least a 20% between-group difference in HCV antibody self-testing between the intervention group and the control group, based on 80% statistical power and an alpha level of 0.05. As this study is one of the first to explore the impact of an HCV self-testing distribution model, there are no references available to draw on for the expected difference in between-group uptake of antibody testing rates. The use of a 20% between-group difference in HCV antibody testing uptake is based on expert opinion, according to previous experiences with HIV self-testing and the increases in the uptake of testing seen with the use of HIV self-testing [15].

For the primary objective, the null hypothesis is that there is no significant difference in the proportion of individuals reporting HCV test results in the intervention versus control groups. The alternative hypothesis is that home-based HCV self-testing in the intervention group will significantly increase the proportion of individuals reporting HCV test results.

The study is not powered to detect significant differences in the secondary objectives. However, similarly to the primary objective, the null hypothesis would be no significant difference between the intervention versus control groups, with the alternative hypothesis being that home-based HCV self-testing will result in significantly higher numbers of HCV-infected individuals being diagnosed, linked to care and treated, making it a cost-effective approach to HCV case-finding and elimination.

#### Recruitment {15}

Building on the lessons learned from the HIV self-testing pilot study, MAC will use social media to promote this study. Promotional materials will be distributed via various social media platforms, including digital fliers and posters (approved by the Medical Research & Ethics Committee, Ministry of Health, Malaysia), as well as online talk shows and videos that will provide basic information about hepatitis C, why testing is important and details about the HCV self-testing study, including information on how to enrol. Participants do not need to be enrolled in the HIV self-testing pilot study to be eligible for this HCV self-testing impact study.

### Assignment of interventions: allocation

#### Sequence generation {16a}

Prior to study enrolment, a list of potential study IDs in ascending numerical order will be generated and randomised by computer to either an intervention or the control group.

#### Concealment mechanism {16b}

Due to the methods of online enrolment and randomisation used, there is no additional concealment mechanism used.

#### Implementation {16c}

**Fig. 1:**
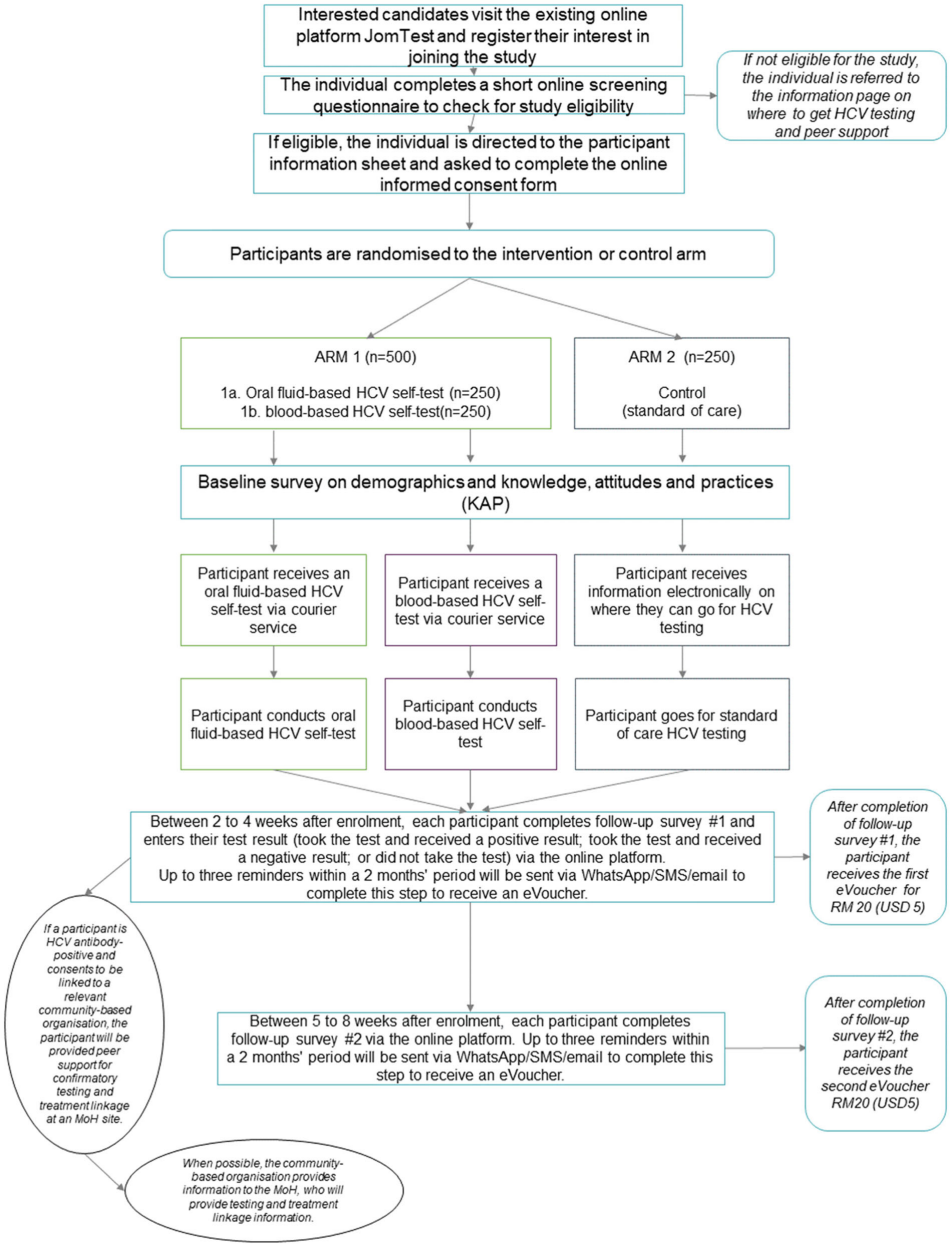
Study flow diagram

As shown in Fig. 1, study participants will be recruited through an existing online platform called JomTest, which currently offers HIV self-testing. Interested participants will sign up and proceed to study eligibility screening; if they are eligible, they will be invited to provide informed consent. After providing consent, enrolled study participants will complete a baseline survey to collect data on their demographics and knowledge of and attitudes towards HCV testing.

Upon enrolment, study participants will be assigned study IDs in a consecutive fashion by the online platform, thereby completing their assignment to a study group. The randomised number encodes a participant’s assignment to one of the study arms, according to the randomisation schedule generated prior to the study by the FIND statistician.

### Assignment of interventions: blinding

#### Who will be blinded {17a}

Participants, researchers and site staff will not be blinded, as this would be impossible due to obvious differences between the two groups (receiving an HCV self-test versus being referred to facility-based testing).

#### Procedure for unblinding if needed {17b}

As this study is not blinded, there is no unblinding procedure.

### Data collection and management

#### Plans for assessment and collection of outcomes {18a}

Participants will complete the baseline, follow-up #1 and follow-up #2 surveys via the online platform (supplementary annex 3). The baseline survey will assess participants’ current knowledge of hepatitis C, including risk factors for contracting hepatitis C, as well as gather information on their current behaviours that may be related to risk factors.

Follow-up survey #1, to be taken 2 to 4 weeks post-enrolment, will ask the participant to report if they have conducted the HCV test and, if so, to provide the results of the test. If the participant reports having taken the test, they will be asked to answer questions relating to their perception of the testing experience and what actions they took following their test result. If the participant reports that they have not yet taken the test, they will be asked questions about why they have not yet taken the test. This survey will also gather information from all participants on their current behaviours that may be related to risk factors for HCV.

Follow-up survey #2, to be taken 4 to 8 weeks post-enrolment, will ask the participant to report if they have taken the HCV test (if they have not already reported taking the test in follow-up survey #1) and, if so, to provide the results of the test. If the participant reports having taken the test, they will be asked to answer questions relating to their perception of the testing experience and what actions they took following their test result. If the participant reports that they have not yet taken the test, they will be asked questions about why they have not yet taken the test. For participants who reported taking the HCV test in follow-up survey #1, follow-up survey #2 will start by gathering information on what actions the person has since taken with regards to seeking further HCV care (if their HCV test was positive). This survey will also gather information from all participants on their current behaviours that may be related to risk factors for HCV.

Data relating to costs will be gathered from a provider’s perspective and will be obtained by conducting a desk review of the costs incurred in the study. These costs will include aspects such as human resources, shipping, and maintaining the online platform.

#### Plans to promote participant retention and complete follow-up {18b}

All participants will be sent a reminder for follow-up survey #1 approximately 2–4 weeks from their day of enrolment. A second follow-up survey will be sent 2 to 4 weeks after the closure of the first survey. Up to three reminders will be sent if a survey has not been completed. To increase the likelihood of participants reporting whether they took the HCV antibody test, participants will receive 20 Malaysian ringgit (~ 5 USD) via electronic voucher or electronic wallet top-ups for completing follow-up survey #1 and an additional 20 Malaysian ringgit for completion of follow-up survey #2.

#### Data management {19}

To ensure information security and confidentiality, the online platform will use verified access to the site, data encryption between personal computers and the website, and encryption of all databases. Information will only be available to users registered with a login and a secure password. Completed informed consent forms and the data recorded via the online platform will be protected with a multilayer security mechanism. Each member of the study team personnel who has access to the files will be assigned appropriate levels of access. Only designated study team personnel will have access to personal identifying information, and these data will not be accessible to the study sponsor.

#### Confidentiality {27}

Participants will be assigned a unique identifier, generated by the study sponsor. Any participant record or dataset that is transferred to the study sponsor will include this identifier only; participant names or any information that would render the participant identifiable will not be transferred.

#### Plans for collection, laboratory evaluation and storage of biological specimens for genetic or molecular analysis in this trial/future use {33}

As there will be no collection of biological specimens for this study, there is no opportunity for genetic or molecular analysis.

## Statistical methods

### Statistical methods for primary and secondary outcomes {20a}

Data will be analysed using R (version 4.1 or higher). A summary of the statistical analyses that will be completed by the endpoint is shown in Table 2. These analyses will be conducted among the group of participants who received the oral fluid-based HCV self-test with the control group and the group of participants who received the blood-based HCV self-test with the control group.

**Table 2:** Statistical analyses to be completed by the endpoint

Endpoint	Statistical analysis methods
1.1 The number and the proportion of participants who report completing the HCV antibody testing in the intervention group 1.2 To assess whether the proportion of participants who report completing the HCV antibody testing in the intervention group is greater than that reported by the participants in the control group by a margin of 20%	Descriptive statistics: Calculation of proportions with 95% CI of the difference in proportions will be evaluated by using the R function prop.test from the “stats” package, which relies on Wilson’s score method. Test of proportions: Two-sided test with a margin of 20%, implemented with the R function “stats::prop.test” with the following hypotheses: *H*_0_: *p*_fo,*I*_ − *p*_fo,*C*_ ≤ 20% *H*_A_: *p*_fo,*I*_ − *p*_fo,*C*_ > 20% where proportions are defined as *p*_*x*,*y*_ [*x* = investigated outcome (fo, pt, ref, trt), *y* = element of {intervention, control}]) comparing the proportion of individuals reporting HCV test results in the intervention versus control groups and between intervention groups. The differences will be reported together with their confidence intervals and the *p*-value related to the statistical comparison mentioned above. The alpha level will be set at 0.025 (2 two-sided tests with an alpha level of 0.05, with the application of Bonferroni correction).
2.1 Number and proportion of HCV antibody-positive individuals made aware of their status in the intervention versus the control group	Descriptive statistics: Statistical testing comparing the numbers of individuals in the intervention versus control groups and between the intervention groups. For details, see the methodology described for endpoint 1.2.
2.2 Number and proportion of HCV antibody-positive individuals who are referred for and complete HCV RNA confirmatory testing in the intervention versus control group	
2.3 Number and proportion of HCV RNA-positive individuals who begin treatment in the intervention versus the control group	
2.4 Analysis of survey responses using proportions and means	Descriptive statistics for survey responses (e.g. proportions, means), as appropriate for the type of response.
2.5 Cost per test completed and cost per person diagnosed (serology, RNA) in the intervention versus control groups	Ingredients-based cost calculation approach.

In a sensitivity analysis, a possible confounding in terms of the time of proband’s actions will be evaluated by analysing the proportions as events by means of Kaplan-Meier analyses with the advantage that dropouts are taken into account as censored values in addition.

### Interim analyses {21b}

There are no interim analyses planned for this study.

### Methods for additional analyses (e.g. subgroup analyses) {20b}

There are no additional analyses planned for this study.

### Methods in analysis to handle protocol non-adherence and any statistical methods to handle missing data {20c}

The primary analysis will be performed using an intention-to-test analysis. The follow-up and incentivisation methods described above will be used in an attempt to minimise missing data.

### Plans to give access to the full protocol, participant-level data and statistical code {31c}

The datasets used and/or analysed during the study can be made available by the corresponding author upon reasonable request and in agreement with the research collaboration and data transfer guidelines of the Medical Review & Ethics Committee (MREC), MoH Malaysia, MAC and the study sponsor.

### Oversight and monitoring

#### Composition of the coordinating centre and trial steering committee {5d}

The principal investigator has overall responsibility for the supervision of the study and medical responsibility for the participants.

The MAC study coordinator ensures the online platform is functioning correctly and that all study procedures are followed.

The MAC monitoring and evaluation officer ensures that the data capture system of the online platform is functioning correctly and is responsible for data quality.

The MAC study team members send out reminders to participants to complete surveys and organise payment of incentives to participants who have completed the surveys.

The MAC peer support team provides support to participants if they have questions or concerns regarding the testing process, assists those participants who have received an HCV antibody-positive result or is interested in linkage to further care (both intervention and control groups).

The study team meets on a weekly basis. While there is no study steering committee, to promote regional cross-learning, there is a stakeholder information sharing group, comprising members from the MoH and HCV programme implementers in neighbouring countries. This stakeholder information sharing group is designed to promote information sharing within the region about the progress of the study, as well as to promote regional cross-learning on best practices for hepatitis C service delivery. This group is scheduled to meet once per quarter during the study.

#### Composition of the data monitoring committee, its role and reporting structure {21a}

There is no data monitoring committee for this study. This decision was based on the lack of adverse events and serious adverse events in the previous HCV self-testing feasibility and acceptability studies completed in Malaysia and six other countries, as well the fact that many large-scale HIV self-testing studies and pilots have been conducted without such committees.

#### Adverse event reporting and harms {22}

**Fig. 2:**
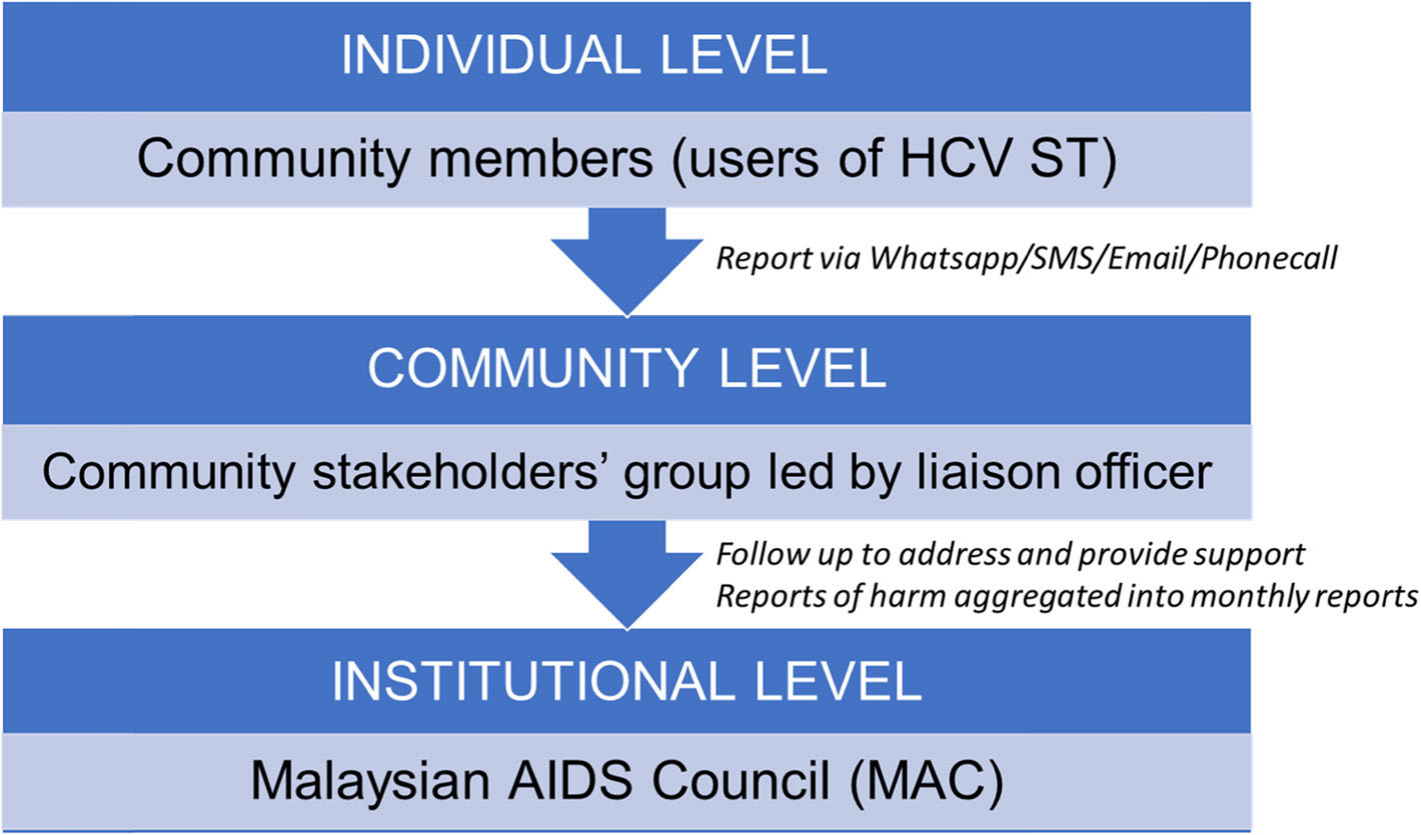
Social harms community stakeholder group

Social harms will be monitored by a community stakeholder group formed by MAC (Fig. 2). The community stakeholder group will follow up any issues and provide support where needed. A telephone hotline will also be made available to participants. Any reports of harm will be aggregated into monthly reports.

#### Frequency and plans for auditing trial conduct {23}

This study will be guided by a risk-based monitoring approach. Before the study begins, it will be verified that the online algorithms are functioning properly regarding informed consent and automatic screening based on eligibility. FIND will create automatic data checks to monitor for unexcepted results, such as more than 1% of respondents in the intervention group reporting an invalid result or being unable to understand the result. Error rates of 1–5% are generally considered acceptable for HIV self-testing [16].

#### Plans for communicating important protocol amendments to relevant parties (e.g. trial participants, ethical committees) {25}

The MREC will be notified of any protocol amendments.

#### Dissemination plans {31a}

The results of this research will be widely disseminated, targeting community groups, academia, implementers and policymakers, via a variety of methods. Participants will receive a lay summary of the results if they opted to do so. A national event to disseminate the study’s findings will be held, which will aim to bring together community and health professionals. The results of the study will also be written up and submitted for presentation at international conferences and publication in peer-reviewed journals.

## Discussion

### Limitations

The study has some important limitations that will be considered and described in the study report. The foremost of these is due to the fact that it is important not to mix different types of tests (oral vs blood) at the same timeframe within the same community which would likely create disruptions and potentially hamper the study, we were unable to enact a 3-arm trial. Rather, the study allocates one type of test kit for the first 250 participants randomised to the intervention group and another type of test kit for the next 250 participants randomised to the intervention group. The control group will be recruited throughout the length of the study to minimise any selection bias over time. Additionally, because enrolment will be conducted via an online platform, the study population will comprise individuals who have access to the Internet and are Internet literate. This may exclude individuals who could also benefit from HCV self-testing but who are unable to access the Internet. Uptake of testing in the control arm may be affected by the geographical location of the participant and the distance to their nearest testing centre. Moreover, the ongoing COVID-19 pandemic could affect the participants’ willingness to visit a healthcare facility, which may have a negative impact on the uptake of testing in the control arm and the uptake of treatment in both the intervention and control arms. Finally, the questionnaires have a multiple-choice design that may not capture some important context-specific aspects.

### Strengths

This study will provide some of the first evidence about the real-world use of HCV self-testing. By using a randomised design, comparing HCV self-testing with facility-based testing among people using a key population-specific online platform, we will provide robust evidence as to whether HCV self-testing increases the uptake of HCV testing in certain stigmatised populations. In addition, in the context of COVID-19-related restrictions on people’s movement, providing home-based HCV self-testing may help to mitigate some of the negative impacts COVID-19 has had on the progress of the national hepatitis programme. This study will also provide an understanding of how the integration of HCV self-testing within existing online platforms for HIV can be used to leverage investments that global funders have made in other areas [17]. This is critical for hepatitis, as currently in the HCV space, there is very limited funding, and most of the funding that is available comes from domestic sources. The findings will also inform the Malaysian MoH programme about HCV self-testing scale-up and help to reach individuals that otherwise may not be reached through existing facility-based services.

## Trial status

Protocol V 3.0 15 December 2021. Enrolment began in September 2021 and is expected be completed by April 2022.

## Supplementary Information

**Additional file 1.**

## Abbreviations

FIND: Foundation for Innovative New Diagnostics
HCV: Hepatitis C virus
HIV: Human immunodeficiency virus
KAP: Knowledge, attitudes and practices
MAC: Malaysian AIDS Council
MREC: Medical Review & Ethics Committee
MoH: Ministry of Health
MSM: Men who have sex with men
RNA: Ribonucleic acid
WHO: World Health Organization

## References

[1] Organization WH (2021). Global progress report on HIV, viral hepatitis and sexually transmitted infections, 2021.

[2] Samarasekera U (2021). Urgent action needed to eliminate viral hepatitis by 2030. Lancet Gastroenterol Hepatol..

[3] WHO (2021). Recommendations and guidance on hepatitis C virus self-testing.

[4] Guise A, Witzel TC, Mandal S, Sabin C, Rhodes T, Nardone A, Harris M (2018). A qualitative assessment of the acceptability of hepatitis C remote self-testing and self-sampling amongst people who use drugs in London, UK. BMC Infect Dis..

[5] Nguyen LT, Nguyen VTT, Le Ai KA, Truong MB, Tran TTM, Jamil MS (2021). Acceptability and usability of HCV self-testing in high risk populations in Vietnam. Diagnostics (Basel).

[6] Martínez-Pérez GZ, Nikitin DS, Bessonova A, et al. Values and preferences for hepatitis C self-testing among people who inject drugs in Kyrgyzstan. BMC Infect Dis. 2021;21:609. 10.1186/s12879-021-06332-z. 10.1186/s12879-021-06332-z PMC8233180 34171990

[7] Majam M, Fischer A, Ivanova Reipold E, Rhagnath N, Msolomba V, Lalla-Edward ST (2021). A lay-user assessment of hepatitis C virus self-testing device usability and interpretation in Johannesburg, South Africa. Diagnostics (Basel).

[8] Reipold EI, Farahat A, Elbeeh A, Soliman R, Aza EB, Jamil MS, Johnson CC, Shiha G, Easterbrook P (2021). Usability and acceptability of self-testing for hepatitis C virus infection among the general population in the Nile Delta region of Egypt. BMC Public Health..

[9] Ingold H, Mwerinde O, Ross AL, Leach R, Corbett EL, Hatzold K, Johnson CC, Ncube G, Nyirenda R, Baggaley RC (2019). The Self-Testing AfRica (STAR) Initiative: accelerating global access and scale-up of HIV self-testing. J Int AIDS Soc.

[10] Md Said R, Mohd Zain R, Chan HK, Soelar SA, Rusli N, Nasir NH, Zakaria R, Hassan MRA (2020). Find the missing millions: Malaysia’s experience with nationwide hepatitis C screening campaign in the general population. J Viral Hepat..

[11] Chan H, Hassali MA, MS R, AHM R (2020). Treatment coverage and drug expenditure in hepatitis C patients from 2013 to 2019: a journey of improving treatment accessibility in Malaysia through government-led initiatives. Hepat Mon.

[12] Malaysia MH (2019). National strategic plan for hepatitis B and C 2019-2023.

[13] Pannir Selvam SB, Khoo EM, Chow SY, Wong PF, Mohsin SS, Abdullah A, Choo WY (2019). Development of a sexually transmitted disease client-friendly unit at a primary care clinic in Malaysia: lessons learnt. Sex Transm Dis..

[14] Jumri N. Antiretroviral treatment: a powerful tool to help HIV patients to lead productive lives, prevent transmissions: UMSC; nd [Available from: https://umsc.my/umsc_news/antiretroviral-treatment-a-powerful-tool-to-help-hiv-patients-to-lead-productive-lives-prevent-transmissions/.

[15] Witzel TC, Eshun-Wilson I, Jamil MS, Tilouche N, Figueroa C, Johnson CC, Reid D, Baggaley R, Siegfried N, Burns FM, Rodger AJ, Weatherburn P (2020). Comparing the effects of HIV self-testing to standard HIV testing for key populations: a systematic review and meta-analysis. BMC Med..

[16] WHO, UNAIDS (2014). A short technical update on self-testing for HIV UNAIDS.

[17] Wingrove C, Hicks J, Regan S, Wang S (2021). Investment cases for hepatitis C: never more important. Lancet Gastroenterol Hepatol..

