# Supplementary material for: A quasi-randomised controlled trial of online distribution of home-based hepatitis C self-testing for key populations in Malaysia: a study protocol
Source: Trials. 2022 Apr 12;23:304. doi: 10.1186/s13063-022-06230-y (PMC9003167; doi:10.1186/s13063-022-06230-y)
Supplement: Supplementary file 1 — Additional file 1. [file 13063_2022_6230_MOESM1_ESM.docx]

**Supplementary Annexes**

**Supplementary Annex 1**

**Screening Questionnaire**

1. What was your sex at birth?
   1. Female
   2. Male

1. What is your age? (*If answered under 18, the questionnaire is stopped, and user is automatically directed to a screen which informs them they are not eligible for the study but provides a list of resources on hepatitis C and how to get testing for hepatitis C)* ____years old
2. Have you been tested for hepatitis C before?
   1. Yes
   2. No
   3. Don’t know

3a. (*Only if answered Yes to question 3)* When were you last tested for hepatitis C virus antibody?

1. Less than 6 months ago (*If answered ‘less than 6 months ago’ the questionnaire is stopped, and user is automatically directed to a screen which informs them they are not eligible for the study but provides a list of resources on hepatitis C and how to get testing for hepatitis C)*
2. More than 6 months ago
3. Do not remember

3b. (*Only if answered Yes to question 3)* What was your hepatitis C virus antibody test result?

1. Positive (*If answered ‘Positive’ the questionnaire is stopped, and user is automatically directed to a screen which informs them they are not eligible for the study and provides a list of resources on hepatitis C including next steps for linkage to care)*
2. Negative
3. Don’t know

1. Are you residing in Malaysia?
   1. No (*If answered ‘No’, the questionnaire is stopped, and user is automatically directed to a screen which informs them they are not eligible for the study and provides a list of resources on hepatitis C)*
   2. Yes

1. Do you identify as belonging to any of the following groups (select all that apply)?
   1. Man who has sex with men
   2. Person who injects drugs
   3. Person who uses drugs
   4. Transgender woman
   5. Sex worker
   6. Person who engages with sex worker
   7. None (*If answered “None”, user is automatically directed to screen which informs them they are not eligible for the study but provides a list of resources on hepatitis C and how to get testing for hepatitis C)*

**Supplementary Annex 2**

**Participant Information Sheet**

**Study Title**

Randomized controlled trial of home-based hepatitis C self-testing for key populations in Malaysia

**Version:** 1.1 dated 10 July 2021

**Participating Organizations:** Ministry of Health (MOH) Malaysia, Malaysian AIDS Council (MAC), FIND

**Sponsor:** FIND, Geneva, Switzerland

**Principal Investigator:** Datuk Dr Muhammad Radzi Abu Hassan

**Introduction**

Hepatitis C is a liver infection caused by a virus that can lead to serious liver damage, cancer, and even death. You are being invited to take part in this study to help understand different ways people can be tested for hepatitis C.

**Purpose**

The purpose of this study is to evaluate hepatitis C self-testing programmes. In order to confirm if you have hepatitis C or not, people currently have to go to a local health clinic to be tested. Sometimes, it is not convenient for the person to travel. We expect to enrol a minimum of 750 participants, aged 18 and above, via an online platform accessible nationwide in Malaysia over the next 6 months. However, for you as an individual, your time in the study will be from when you accept to be part of the study and sign this online form, until you consider getting tested for hepatitis C and complete three online surveys. This usually takes approximately 2 months. The actual amount time you would need to spend completing all the study procedures is approximately 1 to 2 hours.

**Study Procedures**

The time needed to complete each online survey is approximately 20 minutes. You can refuse to answer any question in the online surveys you do not want to answer. Also, you can withdraw from the study at any time and you do not have to give a reason. You will be given a unique number by the online platform and this number randomly determines the following: if you will (1) receive a hepatitis C testing kit delivered to your home/a mailing address you provide or (2) receive information about how to get tested for hepatitis C at a local health clinic. If you receive a hepatitis C testing kit delivered to your home/a mailing address, you will be provided with written and pictorial instructions on how to use the test. If you receive the oral fluid-based test, you will need to swab the test device once along your upper and lower gums and place it in the provided tube for 20 minutes before reading the results. If you receive the blood-based test, you will need to prick your fingertip using the sterile safety needle provided and squeeze to obtain a drop of blood; subsequently, you will need to transfer the blood drop onto the test device, add test buffer and wait for 15 minutes before reading the results. If you receive information about how to get tested for hepatitis C at a local health clinic and seek testing at the clinic, you will be tested by drawing blood from your finger or arm under routine procedures established within the national HCV testing programme, Your information will be reviewed by the study staff and grouped with all other persons in the study.

**Benefits**

As a participant in this study, you may learn if you have been exposed to hepatitis C or not and be offered care and treatment if you have hepatitis C.

**Risks**

There is minimal risk associated with the study. If you receive a blood-based fingerstick HCV testing kit, pricking the finger to take a blood sample may cause minimal bruising, fainting, mild pain or discomfort which is similar to other rapid diagnostic tests used routinely. If you seek HCV testing at a local health clinic, the person who draws your blood is trained. The risks of obtaining blood from your finger or arm may include some temporary discomfort from the needle stick, bruising, or light headedness and fainting. Bruising of this type does not cause long-term problems. The only foreseeable inconvenience associated with the study is the amount of time (1 to 2 hours) you would need to spend completing all the study procedures.

**Compensation and Costs**

It is unlikely that you will suffer any physical harm caused by the oral fluid or blood sample collection procedure. Should this ever occur, MOH will treat any study-related injury since this study is a collaboration with MOH. It will not cost you anything to take part in this study. You will receive RM 20 when you complete Follow-up survey #1 and another RM 20 when you complete Follow-up survey #2. You will be offered hepatitis C testing but are not required to be tested for hepatitis C to receive each compensation. If you receive information about how to get tested for hepatitis C at a local health clinic and seek testing at the clinic, routine procedures established within the national HCV testing programme is free for those eligible. For those not eligible for free testing, you will be charged according to the rates set by MOH.

**Confidentiality**

We will not share any of your personal information outside of the study team. Your name will not be mentioned on the data collected during the study. You will be given a unique number, which will be used to identify the data collected. All your information obtained in this study will be kept and handled in a confidential manner, in accordance with applicable laws and/or regulations. When publishing or presenting the study results, your identity will not be revealed without your expressed consent. Individuals involved in this study, qualified monitors, the sponsor or its affiliates and governmental or regulatory authorities may inspect and copy your medical records, where appropriate and necessary.

You may have the right to access and make a copy of your study records upon writing in to the principal investigator. You may ask to see the records by requesting such records from the principal investigator. However, to ensure the valid results of the study, you may not be able to review or make a copy of some of the records related to the study until after the study has been completed.

Data from the study will be archived for 7 years and may be transmitted outside the country for the purpose of analysis, but your identity will not be revealed at any time. There will not be any remaining or stored specimen from your tests for future studies. You will not be informed of the final study results.

**Voluntary Participation/Withdrawal**

Your participation is voluntary. If you do not want to participate in this study, your access to medical care will not be affected. If you decide to participate, you may drop out from the study at any time without giving a reason and without any penalty or loss of benefits. The study investigator may decide to stop your participation in this study if he/she feels you are not able to continue. The study staff will inform you in a timely manner about any new finding or change which may affect your health or willingness to continue in this study. Where necessary, you may be asked to re-consent to continue to participate in the study.

**Questions**

At any time, if you have any question about the study or want to withdraw from the study, please contact the study team at 011-6267 1992. For questions about your rights as a research participant, please contact: The Secretary, Medical Research & Ethics Committee (MREC), Ministry of Health (MOH) Malaysia at telephone number: 03-3362 8888/8205.

Online Informed Consent Form

**Study title:** Randomized controlled trial of home-based hepatitis C self-testing for key populations in Malaysia

I confirm that I have read and understood the information as provided in the participant information sheet for the above study and have had the opportunity to ask questions.

I understand that the study team may look at my health records and I agree to this access. I understand that my identity will not be revealed in any information released to third parties or published. I understand that I may freely withdraw from this study at any time. I understand I will be given a copy of this consent form via email.

I have understood the study procedures and agree to be a part of the above study.

ð Yes, I consent.

ð No, I do not.

*(Only for those who consented above)*

If you are tested positive for hepatitis C in this study, do you consent for Malaysian AIDS Council (MAC) to contact you and provide you with peer support to access hepatitis C management and care services for confirmatory testing and treatment if required? If you do not consent, it will not affect your participation in this study nor would it affect your access to medical care. On this matter, you can change your mind anytime without giving a reason and without any penalty or loss of benefits.

ð Yes, I consent.

ð No, I do not.

**Supplementary Annex 3**

**PARTICIPANT BASELINE SURVEY**

**STUDY ID: *automatically generated/timestamped by the platform***

**SURVEY DATE:** ***automatically generated/timestamped by the platform***

**INFORMATION TO PARTICIPANTS**

**This questionnaire will be anonymized before being analyzed and your name will never appear in the database.** **Your answers will be used to better understand hepatitis C testing in Malaysia.**

**SECTION A - SOCIODEMOGRAPHICS**

A1. How old are you today?

/ / _/ years old

A2. What was your sex at birth?

1. Male

2. Female

A3. How do you identify your gender?

1. I consider myself male.

2. I consider myself female.

3. I consider myself transgender woman

4. I consider myself transgender man

5. I consider myself non-binary

A4. Which ethnic group do you belong to?

1. Malay

2. Chinese

3. Indian

4. Others, specify:__________

A5. What is your native/primary language?

1. Bahasa Malaysia

2. Mandarin

3. Tamil

4. English

5. Others, specify:__________

A6. What is your employment status?

1. Employed full time
2. Employed part time
3. Unemployed
4. Student
5. Retired
6. Others, specify:__________

A7. What is the highest level of education that you have completed?

1. None

2. Primary

3. Secondary

4. Post-secondary (Certificate, Foundation, Matriculation, STPM, Diploma)

5. University (Bachelor’s degree, Master degree, PhD)

**SECTION B – RISK BEHAVIORS**

B1. In the last 6 months, have you (select all that apply):

1. Had 5 or more sexual partners

2. Been diagnosed with a sexually transmitted infection (HIV, gonorrhea, syphilis, chlamydia, herpes, other)

3. Exchanged sex for money, rent, or other goods

4. Had condomless sex

5. None

B2. How many times have you or your partner(s) used a condom during sexual contact in the last 6 months?

1. I have not had sexual contact in the last 6 months

2. Always

3. Often

4. Sometimes

5. Never used

B3. In the last 6 months, have you taken any substance by snorting it?

1. Yes

2. No

B4. In the last 6 months, have you engaged in chemsex or high-fun/hi fun?

1. Yes

2. No

B5. In the last 6 months, have you injected unprescribed drugs?

1. Once
2. More than once
3. Never

B5b. *(If answered Once or More than once to question B5)* Within the last month, how often did you inject unprescribed drugs?

1. Once a month
2. Several times a month
3. Once a week
4. 2-3 times a week
5. 4-5 times a week
6. Once a day
7. Several times a day
8. Did not inject last month
9. Don’t know

B5c. *(If answered Once or More than once to question B5)* In the past 6 months, have you ever used a needle/syringe that was used by somebody else before?

1. Yes
2. No
3. Don’t know

B5d. *(If answered Yes to question B5c)* If you have used a needle/syringe that was used by somebody else before in the past 6 months, how many people did you share the needle/syringe with?

1. __ (fill in the number of people you shared with)
2. Don’t know

**SECTION C – HEPATITIS C TESTING**

C1. When was the last time you were tested for hepatitis C?

1. No, never tested for hepatitis C

2. Yes, tested for hepatitis C more than 1 year ago

3. Yes, tested for hepatitis C in the past 6 to 12 months

3. Don’t know

C1a. *(If answered Yes, tested for hepatitis C more than 1 year ago or Yes, tested for hepatitis C in the past 6 to 12 months to question C1)* Where were you tested for hepatitis C?

1. Government health clinic
2. Government hospital
3. Private clinic
4. Private hospital
5. Others, specify: _________
6. Don’t know

C1b. *(If answered Never tested for hepatitis C’ to question C1)* Why have you not been tested for hepatitis C (select all that apply)?

1. Do not see myself at risk
2. Do not know how to get tested
3. Have not been interested
4. Do not have time to go to a testing centre
5. Afraid of testing hepatitis C positive
6. Afraid of stigma and/or discrimination if I go to a testing centre and ask for a hepatitis C test
7. Others, specify: __________
8. Don’t know

C2. Where would you most prefer to be tested for hepatitis C?

1. By myself at home
2. At home with someone I trust
3. By myself at a healthcare clinic
4. In a community centre by community-based organization staff
5. In a government healthcare clinic by a healthcare worker
6. In a private clinic by a healthcare worker
7. No preference
8. Prefer not to get tested for hepatitis C
9. Others, specify: ___________

C3. Would you test yourself at home for hepatitis C if you had a testing kit and instructions on how to do it?

1. Yes
2. No
3. Don’t know

**SECTION D – KNOWLEDGE OF HEPATITIS C**

D1. Hepatitis C is caused by a virus.

1. True
2. False
3. Don’t understand
4. Don’t know

D2. Hepatitis C can be transmitted by sex.

1. True
2. False
3. Don’t understand
4. Don’t know

D3. The easiest way to get or give hepatitis C is through deep cuts and sharing needles/syringes.

1. True
2. False
3. Don’t understand
4. Don’t know

D4. Once you are infected with hepatitis C virus, it is possible for you to be a chronic carrier of the disease.

1. True
2. False
3. Don’t understand
4. Don’t know

D5. What can hepatitis C do?

1. Infect cells in the liver
2. Cause inflammation of the liver
3. Cause liver cancer
4. All of the above answers
5. Answers 1 and 2 only
6. Don’t understand
7. Don’t know

D6. Do you know if people can be treated and cured for hepatitis C?

1. Yes, there is a treatment, but not sure about cure
2. Yes, there is treatment and cure
3. Not sure if treatment or cure
4. There is no treatment or cure
5. No idea

D7. Do you know if there is treatment available for hepatitis C in your community/near your community?

1. Yes
2. Yes, but not nearby
3. No
4. No idea

**SECTION E – FINAL SECTION**

E1. *(for intervention group only)*

You will next receive the hepatitis C self-test kit delivered to your home or the address you’ve provided. The kit will include the test, instructions for use, and information about additional supporting materials, such as access to live chat and a call center for questions about testing.

E2. *(for control group only)*

You have been assigned to the control group for this study where you will next receive information about standard of care hepatitis C testing available in your community and additional supporting materials, such as access to live chat and a chat call for questions about testing.

We encourage you to proceed to these facilities for hepatitis C testing.

**PARTICIPANT FOLLOW-UP SURVEY #1**

**STUDY ID: *automatically generated/timestamped by the platform***

**SURVEY DATE:** ***automatically generated/timestamped by the platform***

**INFORMATION TO PARTICIPANTS**

**This questionnaire will be anonymized before being analyzed and your name will never appear in the database.** **Your answers will be used to better understand hepatitis C testing in Malaysia.**

**SECTION A – STUDY TESTING AND FOLLOW-UP**

A1. Did you complete the hepatitis C testing that was offered to you as part of this study?

1. Yes

2. No

A1ai. *(if answered Yes to question A1, version of question for control group)* What was the result?

1. Positive
2. Negative
3. Have not been told the results yet
4. Don’t know, have forgotten
5. Do not want to disclose

A1aii. *(if answered Yes to question A1, version of question for intervention group)* What was the result?

1. Positive
2. Negative
3. Invalid
4. Don’t know
5. Do not want to disclose

A1bi. (*If answered No to question A1, version of question for control group)* If no, why not?

1. Did not want to test/was not interested
2. Forgot to get tested
3. Afraid of testing
4. Did not have time
5. Test was too expensive
6. Others, specify: __________

A1bii. (*If answered No to question A1, version of question for intervention group)* If no, why not?

1. Did not want to test/was not interested
2. Forgot to get tested
3. Afraid of testing
4. Did not understand how to do the test
5. Did not have time
6. Others, specify: __________

A1c. *(If answered Yes for question A1, for control group only)* Where did you go to get the hepatitis C test done?

_____________ (fill in name of place you got the testing at)

A1f. *(If answered Negative in question A1aii, for intervention group only)* If you had tested positive for hepatitis C, what do you think your next steps would have been?

1. To go to a community-based organization for more information and advice
2. To go to a government healthcare clinic for a confirmation test
3. To go to a private clinic for a confirmation test
4. Don’t know
5. Others, specify: ___________

A1g. *(If answered Invalid or Don’t know in question A1aii, for intervention group only)* Have you taken any further step to get a second test done?

1. Yes, have gone to a community-based organization for more information and advice
2. Yes, have gone to a government healthcare clinic and asked for another test
3. Yes, have gone to a private clinic and asked for another test
4. Others, specify: ___________
5. No

A1h. *(If answered No in question A1g)* If no, why not?

1. Did not want to test/was not interested
2. Forgot to get tested
3. Afraid of testing
4. Did not have time
5. Test was too expensive
6. Others, specify: __________

A2a. (*version of question for control group)* Did you ask anyone any question about hepatitis C testing?

1. Yes, online through the support offered on JomTest platform
2. Yes, online through searching the internet
3. Yes, person who performed the test
4. Yes, friend or family member
5. Yes; others, specify: ______________
6. No

A2b. (*version of question for intervention group)* Did you ask anyone any question about hepatitis C testing?

1. Yes, online through the support offered on JomTest platform
2. Yes, online through searching the internet
3. Yes, friend or family member
4. Yes; others, specify: ______________
5. No

A3. *(If answered Yes in question A1)* How would you rate the hepatitis C testing you were offered in each of the following categories?

Not very easy Average Very easy

How easy was the testing process? 1 2 3 4 5

Not very convenient Average Very convenient

How convenient was the testing process? 1 2 3 4 5

Not very private Average Very private

How private did you think the testing process was? 1 2 3 4 5

Not very trustworthy Average Very trustworthy

How much do you feel you can trust the test results? 1 2 3 4 5

Not very secure Average Very secure

How secure did you feel during the testing process? 1 2 3 4 5

Not very stressful Average Very stressful

How stressful was the testing process? 1 2 3 4 5

Not very easy Average Very easy

If you needed further care, how easy was it to access it? 1 2 3 4 5 Did not need it

A4. *(If answered Yes in question A1)* Did you feel you could understand the result of your test?

1. Yes
2. No

A4ai. *(If answered Yes in question A4, version of question for intervention group)* What do you think have helped you to understand the result of your test (select all that apply)?

1. The printed instructions for use that came with the HCV self-test
2. Video instructions on how to perform a self-test
3. Being able to communicate with the JomTest team
4. Others, specify: ____________

A4aii. *(If answered No in question A4, version of question for intervention group)* Why do you think you were unable to understand the result of your test? Select all that apply

1. The printed instructions for use that came with the HCV self-test were not easy to understand
2. Video instructions on how to perform a self-test was not easy to understand
3. Communication with the JomTest team were not easy to understand
4. Others; specify: ___________

A5. *(If answered Positive, Invalid or Don’t know in question A1aii)* Did you feel you knew what steps you needed to take to be further linked to hepatitis C care after you got the result of your test?

1. Yes
2. No

A6. *(If answered No in question A5)* What do you think would have helped you to know what steps you need to take to be further linked to care?

1. A list of clinics near me that provide HCV care with their contact information
2. More information on how community-based organizations near me could help me navigate how to be linked to care
3. A video explaining how I could get linked to care
4. Others; specify:_________

A7. In the future, where would you prefer to be tested for hepatitis C?

1. By myself at home
2. At home with someone I trust
3. By myself at a community center
4. In a community centre by community-based organization staff
5. In a government healthcare clinic by a healthcare worker
6. In a private clinic by a healthcare worker
7. No preference
8. Prefer not to get tested for hepatitis C
9. Others, specify: __________

A8. In the future, would you test yourself at home if you have a hepatitis C self-testing kit and instructions on how to do it?

1. Yes
2. No
3. Don’t know

A8a. *(If answered Yes in question A8)* If yes, how often do you think you would test yourself?

1. More than once every 6 months
2. Once every 6 months
3. Once a year
4. Once every 2 years
5. Don’t know
6. Others, specify:_______________

A1d. *(If answered Positive in question A1ai or A1aii)* Have you taken further steps for hepatitis C care after your positive test?

1. Yes, have gone for confirmation test
2. Yes, have completed further testing and have started treatment
3. Yes; others, specify: ___________
4. No

A1e. *(If answered Yes, have gone for confirmation test or Yes, have completed further testing and have started treatment in question A1d)* What was the result of your confirmation test?

1. I have hepatitis C viremia
2. I do not have hepatitis C viremia
3. Have not been told the results yet
4. Others, specify:__________

A1i. *(If answered Yes, have gone for confirmation test or Yes, have completed further testing and have started treatment in question A1d)* Where did you go for this further hepatitis C care?

_____________ (fill in name of place you went for further hepatitis C care at)

**SECTION B – RISK BEHAVIORS**

B1. How many times have you or your partner(s) used a condom during sexual contact in the last month?

1. I have not had sexual contact in the last month

2. Always

3. Often

4. Sometimes

5. Never used

B2. In the last month, have you taken any substance by snorting it?

1. Yes

2. No

B3. In the last month, have you engaged in chemsex or high-fun/hi-fun?

1. Yes

2. No

B4. In the last month, have you injected unprescribed drugs?

1. Once
2. More than once
3. Never

B4a. (*If answered Once or More than once to question B4)* Within the last month, how often did you inject drugs?

1. Once a month
2. Several times a month
3. Once a week
4. 2-3 times a week
5. 4-5 times a week
6. Once a day
7. Several times a day
8. Don’t know

B4b. (*If answered Once or More than once to question B4)* In the past month, have you ever used a needle/syringe that was used by somebody else before?

1. Yes
2. No
3. Don’t know

B4c. (*If answered Yes to question B4b)* If you have used a needle/syringe that was used by somebody else before in the past month, how many people did you share the needle/syringe with?

1. __ (fill in the number of people you shared with)
2. Don’t know

**SECTION C – Help us to make hepatitis C testing accessible to everyone who needs it, your opinion counts!**

Please let us know how we can improve hepatitis C testing and care services - your feedback will help to guide how these services can best serve the people in Malaysia:

____________________________________________________________________________

**PARTICIPANT FOLLOW-UP SURVEY #2**

**STUDY ID: *automatically generated/timestamped by the platform***

**SURVEY DATE:** ***automatically generated/timestamped by the platform***

**INFORMATION TO PARTICIPANTS**

**This questionnaire will be anonymized before being analyzed and your name will never appear in the database.** **Your answers will be used to better understand hepatitis C testing in Malaysia.**

**SECTION A – STUDY TESTING AND FOLLOW-UP**

A1. *(If answered No to question A1 in Follow-up survey #1)* Did you complete the hepatitis C testing that was offered to you as part of this study?

1. Yes

2. No

A1ai. *(if answered Yes to question A1, version of question for control group)* What was the result?

1. Positive
2. Negative
3. Have not been told the results yet
4. Don’t know, have forgotten
5. Do not want to disclose

A1aii. *(if answered Yes to question A1, version of question for intervention group)* What was the result?

1. Positive
2. Negative
3. Invalid
4. Don’t know
5. Do not want to disclose

A1bi. (*If answered No to question A1, version of question for control group)* If no, why not?

1. Did not want to test/was not interested
2. Forgot to get tested
3. Afraid of testing
4. Did not have time
5. Test was too expensive
6. Others, specify: __________

A1bii. (*If answered No to question A1, version of question for intervention group)* If no, why not?

1. Did not want to test/was not interested
2. Forgot to get tested
3. Afraid of testing
4. Did not understand how to do the test
5. Did not have time
6. Others, specify: __________

A1c. *(If answered Yes for question A1 for control group only)* Where did you go to get the hepatitis C test done?

_____________ (fill in name of place you got the testing at)

A1f. *(If answered Negative in question A1aii, for intervention group only)* If you had tested positive for hepatitis C, what do you think your next steps would have been?

1. To go to a community-based organization for more information and advice
2. To go to a government healthcare clinic for a confirmation test
3. To go to a private clinic for a confirmation test
4. Don’t know
5. Others, specify: ___________

A1g. *(If answered Invalid or Don’t know in question A1aii, for intervention group only)* Have you taken any further step to get a second test done?

1. Yes, have gone to a community-based organization for more information and advice
2. Yes, have gone to a government healthcare clinic and asked for another test
3. Yes, have gone to a private clinic and asked for another test
4. Others, specify: ___________
5. No

A1h. *(If answered No in question A1g)* If no, why not?

1. Did not want to test/was not interested
2. Forgot to get tested
3. Afraid of testing
4. Did not have time
5. Test was too expensive
6. Others, specify: __________

A2a. (*version of question for control group)* Did you ask anyone any question about hepatitis C testing?

1. Yes, online through the support offered on JomTest platform
2. Yes, online through searching the internet
3. Yes, person who performed the test
4. Yes, friend or family member
5. Yes; others, specify: ______________
6. No

A2b. (*version of question for intervention group)* Did you ask anyone any question about hepatitis C testing?

1. Yes, online through the support offered on JomTest platform
2. Yes, online through searching the internet
3. Yes, friend or family member
4. Yes; others, specify: ______________
5. No

A3. *(If answered Yes in question A1)* How would you rate the hepatitis C testing you were offered in each of the following categories?

Not very easy Average Very easy

How easy was the testing process? 1 2 3 4 5

Not very convenient Average Very convenient

How convenient was the testing process? 1 2 3 4 5

Not very private Average Very private

How private did you think the testing process was? 1 2 3 4 5

Not very trustworthy Average Very trustworthy

How much do you feel you can trust the test results? 1 2 3 4 5

Not very secure Average Very secure

How secure did you feel during the testing process? 1 2 3 4 5

Not very stressful Average Very stressful

How stressful was the testing process? 1 2 3 4 5

Not very easy Average Very easy

If you needed further care, how easy was it to access it? 1 2 3 4 5 Did not need it

A4. *(If answered Yes in question A1)* Did you feel you could understand the result of your test?

1. Yes
2. No

A4ai. *(If answered Yes in question A4, version of question for intervention group)* What do you think have helped you to understand the result of your test (select all that apply)?

1. The printed instructions for use that came with the HCV self-test
2. Video instructions on how to perform a self-test
3. Being able to communicate with the JomTest team
4. Others, specify: ____________

A4aii. *(If answered No in question A4, version of question for intervention group)* Why do you think you were unable to understand the result of your test? Select all that apply

1. The printed instructions for use that came with the HCV self-test were not easy to understand
2. Video instructions on how to perform a self-test was not easy to understand
3. Communication with the JomTest team were not easy to understand
4. Others; specify: ____________

A5. *(If answered Positive, Invalid or Don’t know in question A1aii)* Did you feel you knew what steps you needed to take to be further linked to hepatitis C care after you got the result of your test?

1. Yes
2. No

A6. *(If answered No in question A5)* What do you think would have helped you to know what steps you need to take to be further linked to care?

1. A list of clinics near me that provide HCV care with their contact information
2. More information on how community-based organizations near me could help me navigate how to be linked to care
3. A video explaining how I could get linked to care
4. Others; specify:_________

A7. In the future, where would you prefer to be tested for hepatitis C?

1. By myself at home
2. At home with someone I trust
3. By myself at a community center
4. In a community centre by community-based organization staff
5. In a government healthcare clinic by a healthcare worker
6. In a private clinic by a healthcare worker
7. No preference
8. Prefer not to get tested for hepatitis C
9. Others, specify: ___________

A8. In the future, would you test yourself at home if you have a hepatitis C testing kit and instructions on how to do it?

1. Yes
2. No
3. Don’t know

A8a. *(If answered Yes in question A8)* If yes, how often do you think you would test yourself?

1. More than once every 6 months
2. Once every 6 months
3. Once a year
4. Once every 2 years
5. Don’t know
6. Others, specify:_______________

A1d. *(If answered Yes to question A1, Positive in question A1a and No in question A1d in Follow-up survey #1, this will be the first question for them in this Follow-up survey #2. After questions A1d and A1e have been answered by this group in Follow-up survey #2, they will proceed to section B. This question is also for those who answered Positive in question A1a in Follow-up survey #2; for this group, they will proceed through the rest of section A following skip patterns based on their answers)* Have you taken further steps for hepatitis C care after your positive test?

1. Yes, have gone for confirmation test
2. Yes, have completed further testing and have started treatment
3. Yes; others, specify: ___________
4. No

A1e. *(If answered Yes, have gone for confirmation test or Yes, have completed further testing and have started treatment in question A1d)* What was the result of your confirmation test?

1. I have hepatitis C viremia
2. I do not have hepatitis C viremia
3. Have not been told the results yet
4. Others, specify:__________

A1i. *(If answered Yes, have gone for confirmation test or Yes, have completed further testing and have started treatment in question A1d)* Where did you go for this further hepatitis C care?

_____________ (fill in name of place you went for further hepatitis C care at)

**SECTION B – RISK BEHAVIORS**

B1. How many times have you or your partner(s) used a condom during sexual contact in the last month?

1. I have not had sexual contact in the last month

2. Always

3. Often

4. Sometimes

5. Never used

B2. In the last month, have you taken any substance by snorting it?

1. Yes

2. No

B3. In the last month, have you engaged in chemsex or high-fun/hi-fun?

1. Yes

2. No

B4. In the last month, have you injected unprescribed drugs?

1. Once
2. More than once
3. Never

B4a. (*If answered Once or More than once to question B4)* Within the last month, how often did you inject drugs?

1. Once a month
2. Several times a month
3. Once a week
4. 2-3 times a week
5. 4-5 times a week
6. Once a day
7. Several times a day
8. Don’t know

B4b. (*If answered Once or More than once to question B4)* In the past month, have you ever used a needle/syringe that was used by somebody else before?

1. Yes
2. No
3. Don’t know

B4c. *(If answered Yes to question B4b)* If you have used a needle/syringe that was used by somebody else before in the past month, how many people did you share the needle/syringe with?

1. __ (fill in the number of people you shared with)
2. Don’t know

**SECTION C – Help us to make hepatitis C testing accessible to everyone who needs it, your opinion counts!**

Please let us know how we can improve hepatitis C testing and care services - your feedback will help to guide how these services can best serve the people in Malaysia:

____________________________________________________________________________
